# Effects of sevoflurane postconditioning on cell death, inflammation and TLR expression in human endothelial cells exposed to LPS

**DOI:** 10.1186/1479-5876-11-87

**Published:** 2013-04-03

**Authors:** Raquel Rodríguez-González, Aurora Baluja, Sonia Veiras Del Río, Alfonso Rodríguez, Jaime Rodríguez, Manuel Taboada, David Brea, Julián Álvarez

**Affiliations:** 1 Critical Patient Translational Research Group, Department of Anesthesiology, Intensive Care and Pain Management, Hospital Clínico Universitario, IDIS, University of Santiago de Compostela, Santiago de Compostela, Spain; 2 Research Unit, Hospital Universitario Dr. Negrín, Las Palmas de Gran Canaria, Spain; 3 CIBER de Enfermedades Respiratorias, Instituto de Salud Carlos III, Madrid, Spain; 4 Clinical Neurosciences Research Laboratory, Department of Neurology, Hospital Clínico Universitario, University of Santiago de Compostela, Santiago de Compostela, Spain; 5 Cellular and Molecular Neurobiology Research Group and Grup de Recerca en Neurociencies del IGTP, Department of Neurosciences, Fundació Institut d'Investigació en Ciències de la Salut Germans Trias I Pujol-Universitat Autónoma de Barcelona, Badalona, Spain

**Keywords:** Sevoflurane, Endothelium, Inflammation, TLR, Sepsis, Postconditioning

## Abstract

**Background:**

Sevoflurane is an anesthetic agent which also participates in protective mechanisms in sepsis, likely due to anti-inflammatory properties. A key tissue in sepsis is the endothelium, which expresses TLR2 and TLR4 receptors, known regulators of inflammatory mechanisms and potential therapeutic targets for this pathology. In this context, we explored the effect of sevoflurane postconditioning in an *in vitro* sepsis model.

**Methods:**

Primary cultures of human umbilical vein endothelial cells were used for two different experiments. In the first set, cultures were placed in an airtight incubation chamber and exposed to different concentrations of sevoflurane (0,1,3 or 7% vol,) for 1 hour. In the second set, lipopolysaccharide from *Escherichia coli* 0111:B4 (1 μg/mL) was added to culture medium for 3 hours and cells were subsequently exposed to sevoflurane (0,1,3 or 7% vol,) for 1 hour as explained before. In both cases, cell viability was measured by MTT and Trypan blue assays, TLR2 and TLR4 expression were analyzed by flow cytometry, and TNFα and IL-6 levels were quantified in cell culture media by an immunoassay immediately after exposure, at 6 and 24 hours.

**Results:**

Exposure to 3% sevoflurane decreased TLR2 at 24 hours and TLR4 at 6 and 24 hours (both p<0.05), whereas exposure to 7% decreased TLR4 expression at 6 hours (p<0.05). Both 3 and 7% sevoflurane decreased TNF-α and IL-6 levels at 24 hours (both p<0.05). In LPS-stimulated cultures, exposure to 3% sevoflurane was cytoprotective at 6 and 24 hours (p<0.05) compared with control, and decreased TLR2 and TLR4 expression at 24 hours (p<0.05); whereas 7% decreased TLR4 expression at 24 hours (p<0.05). Both 3% and 7% sevoflurane decreased TNF-α and IL-6 levels at 24 hours (both p<0.05).

**Conclusions:**

Postconditioning with the halogenated anesthetic agent sevoflurane after LPS stimulation shows a cytoprotective effect in an *in vitro* model, decreasing cell death and reducing TLR2 and TLR4 expression as well as levels of the inflammatory mediators TNF-α and IL-6 in human endothelial cells.

## Introduction

Sepsis remains a major clinical problem due to high morbidity and mortality with limited therapeutic options, being the leading cause of death in non-coronary intensive care units and an important burden for healthcare resources. A crucial tissue involved in sepsis pathogenesis is the endothelium, cells involved in hemodynamics, immunity and coagulation pathways, the three main cornerstones of septic response [1].

Sevoflurane is a highly fluorinated methyl-isopropyl ether widely used for induction and maintenance of general anesthesia. In addition to its anesthetic properties, it has also shown to be involved in protective mechanisms in conditions of hypoxia or endotoxemia, mostly studied in neuronal and myocardial tissues [2-4]. A study by Kidani et al. examined the effects of sevoflurane pretreatment on mortality and inflammation during endotoxin-induced shock in rats. Importantly, they found that this pretreatment significantly improved systolic blood pressure, acid–base balance and reduced mortality rates and plasma levels of TNF-α and IL-6, thus showing an attenuation of the inflammatory response [5].

In addition to preconditioning, it has been demonstrated that sevoflurane induces a postconditioning effect after exposure to hypoxia or lipopolysaccharide (LPS). In this regard, it has been demonstrated that sevoflurane postconditioning decreases blood and brain oxidative injury and enhances immunity indexes in rats subjected to cerebral ischemia-reperfusion [6]. Importantly, data from Yue et al. analyzing sevoflurane postconditioning in an *in vitro* model of acute lung injury show that it significantly reduced inflammatory mediators, chemotaxis and neutrophil adherence [7]. Therefore, evidence from experimental data support the idea of sevoflurane postconditioning as an organ-protective strategy.

Bacterial infection initiates a series of responses that contribute to endothelial dysfunction, resulting in fluid leakage, platelet adherence and the release of adhesion molecules and inflammatory mediators, among others. Consequently, endothelial cells are key players in sepsis, expressing toll-like receptor (TLR) 4 and TLR2, essential components of the innate immune system and pathogen recognition mechanisms. TLR-mediated signaling cascade triggered by bacterial ligands result in the activation of NF-κB, leading to the transcription of a range of important pro-inflammatory cytokine and chemokine genes, such as TNFα, IL-1, IL-6, IL-12 and IL-8 [8-11], playing an important role in endothelial inflammation. Importantly, TLRs are dynamically modulated across the stages of sepsis, and experimental data indicate that, by dampening TLR-induced inflammatory pathways, it is possible to interfere with the progression of sepsis [12,13].

Due to demonstrated anti-inflammatory effects of sevoflurane preconditioning in sepsis, we aimed to investigate the possible effect of sevoflurane postconditioning in an *in vitro* model of endotoxaemia using LPS-exposed human endothelial cells, a highly dynamic tissue responsive to exposure to this anesthetic agent [14], focusing on the interaction between sevoflurane and TLR expression, as inductors of LPS-mediated cytokine response.

## Methods

### Cell culture

Human endothelial cells (HUVECs) were kindly provided by Dr. E. Álvarez (Instituto de Investigación Sanitaria de Santiago de Compostela (IDIS), Spain). Cells were isolated from freshly obtained human umbilical cords donated under informed consent of the mothers by following the method previously described [15]. All procedures were approved by the Ethics Committee for Clinical Research in Galicia (Spain), according to the World Medical Association Declaration of Helsinki. After dissociation, HUVEC were cultured on 0.2% gelatin-coated flasks (BD Biosciences, Madrid, Spain) using endothelial cell growth medium (Promocell, Heidelberg, Germany,) and grown to confluence in an incubator at 37°C with a humidified atmosphere containing 95% air/5% CO_2._ Cells were expanded by trypsinization with 0.25% trypsin in PBS containing 0.025% EDTA (Sigma, Madrid, Spain). For the experiments, cells were used between the second and fifth passage and seeded at a density of 10^5^cells per cm^2^ in multiwell plates (BD Biosciences, Madrid, Spain). Medium was replaced every 3 days by fresh growth medium and cells were allowed to reach confluence.

### Sevoflurane exposure

To analyze the possible effect of sevoflurane on different parameters, cultures were exposed to this anesthetic agent mainly as previously described [16,17]. Endothelial cells were exposed to three different concentrations of sevoflurane (Sevorane®, Abbott Laboratories, Madrid, Spain): 1, 3, or 7 vol.% by means of a vaporizer (Abbott) for 1 hour. Cell cultures were placed in an airtight incubation chamber (Billups-Rothenberg, Del Mar, CA) at a constant temperature of 37°C and subsequently perfused with air (21% O_2_, 5% CO_2,_ 69% N_2_) containing 1, 3 or 7% sevoflurane. These doses have been selected in accordance to the clinical context, where sevoflurane doses with anesthetic purposes range from 3% vol (induction) to 5-8% vol (maintenance). After 12 minutes with a flow rate of 4 L/min, sevoflurane concentrations in the chamber remained stable. Gas concentrations of O_2_, CO_2_ and sevoflurane were continuously monitored from the output line by an anesthetic gas measurer module (Capnomac Ultima, Datex Ohmeda, GE Healthcare, Madrid, Spain), showing no sevoflurane loss in the circuit and remaining steady during the exposure time. For each condition, control cells were superfused for the same length of time with fresh air lacking sevoflurane. Once exposure time was finished, cells were returned to the incubator. In order to evaluate the temporal profile of the analyzed molecules, samples of culture media and cells were taken from both treated and control groups immediately after finishing exposure (t=0), at 6 (t=6) and 24 (t=24) hours after sevoflurane exposure. All experiments were carried out three times in triplicate.

### LPS treatment

In a different set of experiments, we aimed to analyze sevoflurane-mediated cytoprotection after LPS treatment in human endothelial cells. Pilot experiments testing increasing doses of LPS were carried out in order to determine the sub-lethal LPS concentration that activates HUVEC in our cultures, resulting this to be 1 μg/mL (data not shown). Therefore, 1 μg/mL LPS from *Escherichia coli* 0111:B4 (Sigma) was added to the culture medium and cells were returned to the incubator. Three hours after LPS stimulation, cell cultures were exposed to sevoflurane for 1 hour as explained above (1, 3 or 7% vol.% sevoflurane) in order to evaluate the postconditioning effect of this anesthetic agent. Control cells were treated with LPS but were not exposed to sevoflurane. Samples of culture media and cells for analysis were taken from both treated and control groups immediately after finishing sevoflurane exposure (t=0), at 6 (t=6) and 24 (t=24) hours (see experimental protocol scheme in Additional file 1). Experiments were conducted in triplicate.

### MTT assay

Cell viability was analyzed by the colorimetric 3-(4,5-dimethylthiazol-2-yl)-2,5-diphenyl tetrazolium bromide (MTT) assay carried out in cell samples collected just after exposure (t=0), at 6 (t=6) and 24 (t=24) hours after, using a commercial kit (Sigma) and a microplate reader (Biotek) for spectrophotometric measurements.

### Trypan blue exclusion assay

Cell death was also determined by Trypan blue dye exclusion test. The assay is based on the fact that the dye is negatively charged and does not interact with the cell unless the membrane is damaged. At the mentioned timepoints, cells from each group were detached by trypsinization and subsequently collected by centrifugation. After resuspension in phosphate buffered saline, equal volumes of cell suspension and 0.4% Trypan blue (Invitrogen) were mixed and incubated for 10 min at room temperature. The number of dead cells (blue stained cells) was counted using a hemocytometer from five random fields using a counting grid. Data are expressed as percent of total cells/field ± SD.

### Flow cytometry analysis of TLR2 and TLR 4

Expression of TLR2 and TLR4 on endothelial cells was analyzed by flow cytometry at the mentioned timepoints. APC-TLR2 antibody (BD Biosciences) and biotin-TLR4 antibody (BD Biosciences) together with streptavidin-PE (BD Biosciences) were used to quantify TLR expression. Samples were analyzed on a FACSAria flow cytometer (BD Biosciences) [18]. Mean expression of TLR2 and TLR4 on HUVEC was analyzed and expressed as AFU (arbitrary fluorescence units).

### Determination of TNF-α and IL-6

TNF-α and IL-6 were measured in the culture media of all groups collected at the mentioned timepoints (t=0, t=6 and t=24) after sevoflurane exposure using an immunoassay-based chemiluminescent automated system (Immulite 1000, Siemens Healthcare, Madrid, Spain). Inter- and intra assay coefficients of variation were below 6% for both molecules.

### Statistical analysis

Results are expressed as mean ± standard deviation (SD) of the indicated number of experiments, and statistical analysis was made using Student’s *t* test with SPSS 14.0 software. *P* values <0.05 were considered statistically significant.

## Results

### Effects of sevoflurane exposure on endothelial cultures

**Figure 1:**
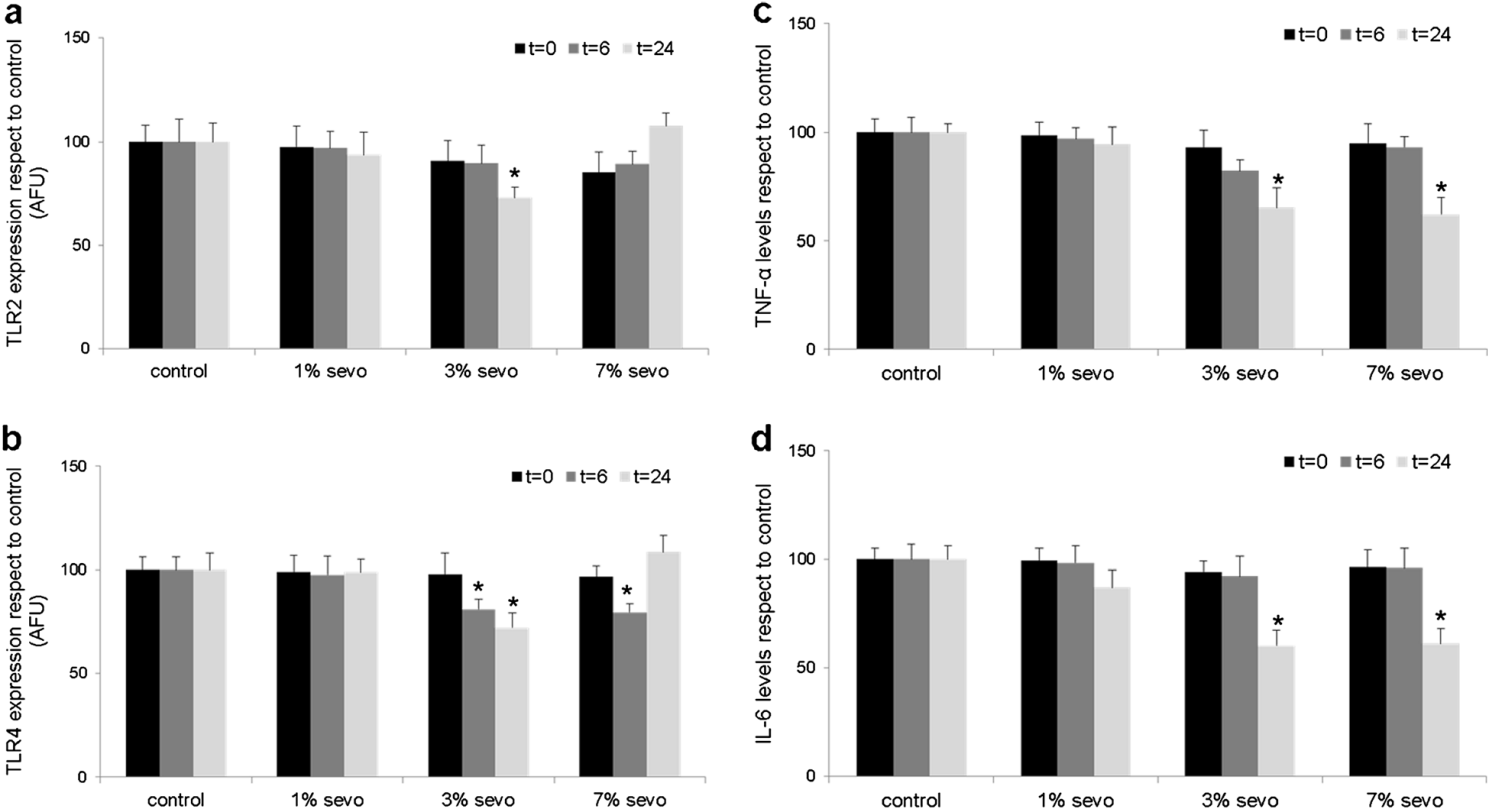
**Effect of different concentrations of sevoflurane on HUVEC cultures at different timepoints.** (**a**) Effect on TLR2 expression; (**b**) effect on TLR4 expression; (**c**) levels of TNF-α (**d**) and IL-6. * p <0.05. Analysis was performed immediately after finishing sevoflurane exposure (t=0), at 6 (t=6) and 24 (t=24) hours. AFU: Arbitrary fluorescence units.

Sevoflurane exposure did not induce cell death in our cultures at any studied concentration or timepoint, as revealed by both MTT and Trypan blue assays (see Additional file 1), and no morphological alterations were found (data not shown). However, flow cytometry analysis showed sevoflurane-induced changes in TLR2 and TLR4 expression. Exposure to 3% sevoflurane lowered TLR2 expression at all analyzed timepoints, being this decrease statistically significant at 24 hours after exposure compared to control (Figure 1a, p<0.05, n=9). With respect to TLR4 expression, it was reduced by 3% sevoflurane at 6 and 24 hours after exposure, while 7% sevoflurane only showed a significant reduction at 6 hours compared to control (Figure 1b, all p<0.05, n=9). Because TLR2 and TLR4 signalling cascades trigger inflammation, and given the relationship found between sevoflurane and TLRs in the present study, we also measured levels of the inflammatory cytokines TNF-α and IL-6 in our culture. These results show that exposure to 1% sevoflurane did not affect cytokine levels, whereas sevoflurane at 3% and 7% significantly lowered both TNF-α and IL-6 levels in culture media at 24 hours (Figure 1c,d, both p<0.05, n=9).

### Effect of sevoflurane exposure after LPS treatment on endothelial cultures

**Figure 2:**
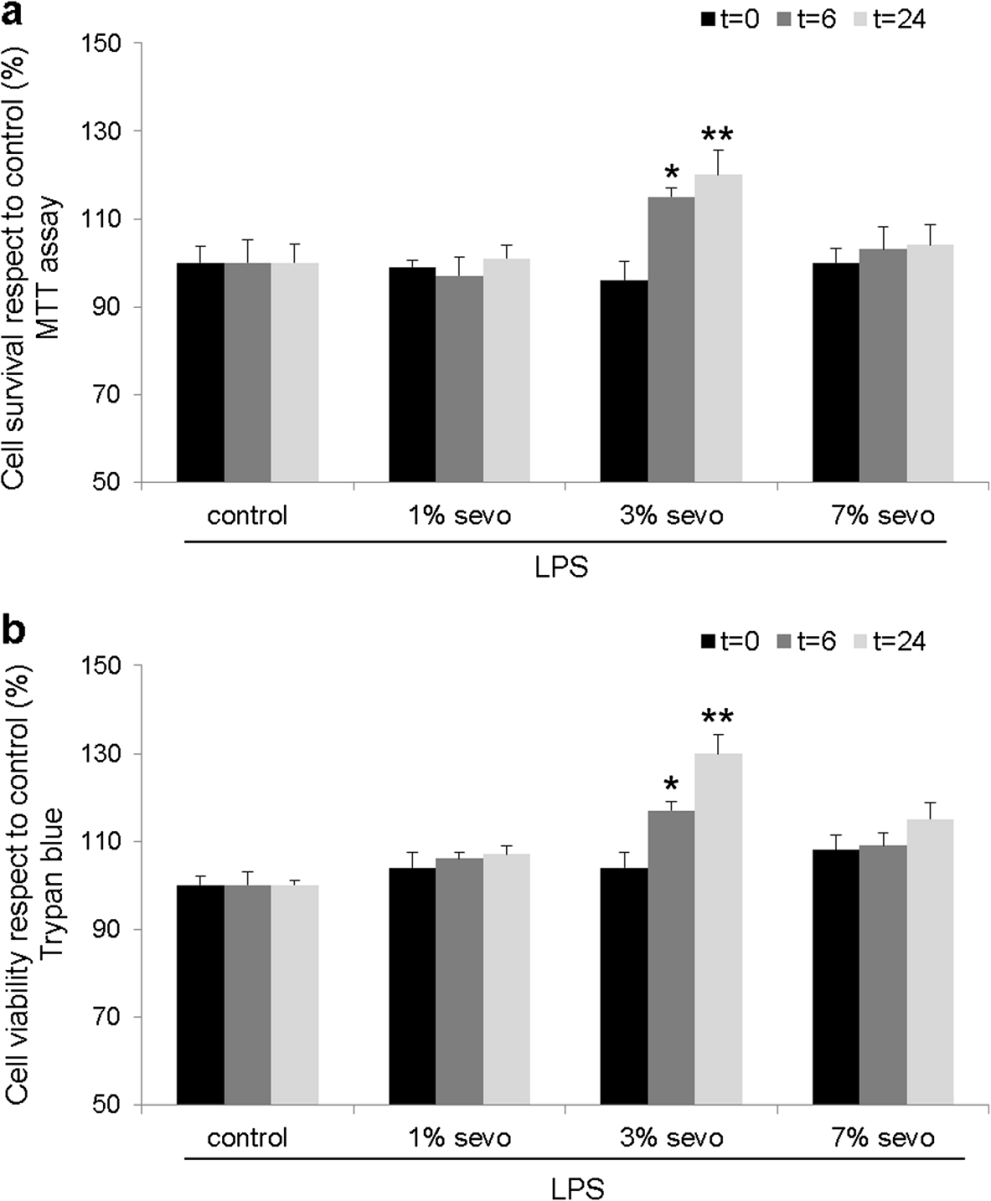
**Cytoprotective effect of sevoflurane postconditioning on HUVEC after LPS stimulation measured by MTT (a) and Trypan blue (b) assays.** * p<0.05, ** p<0.01. Analysis was performed immediately after sevoflurane exposure (t=0), at 6 (t=6) and 24 (t=24) hours. LPS: Lipopolysaccharide.

**Figure 3:**
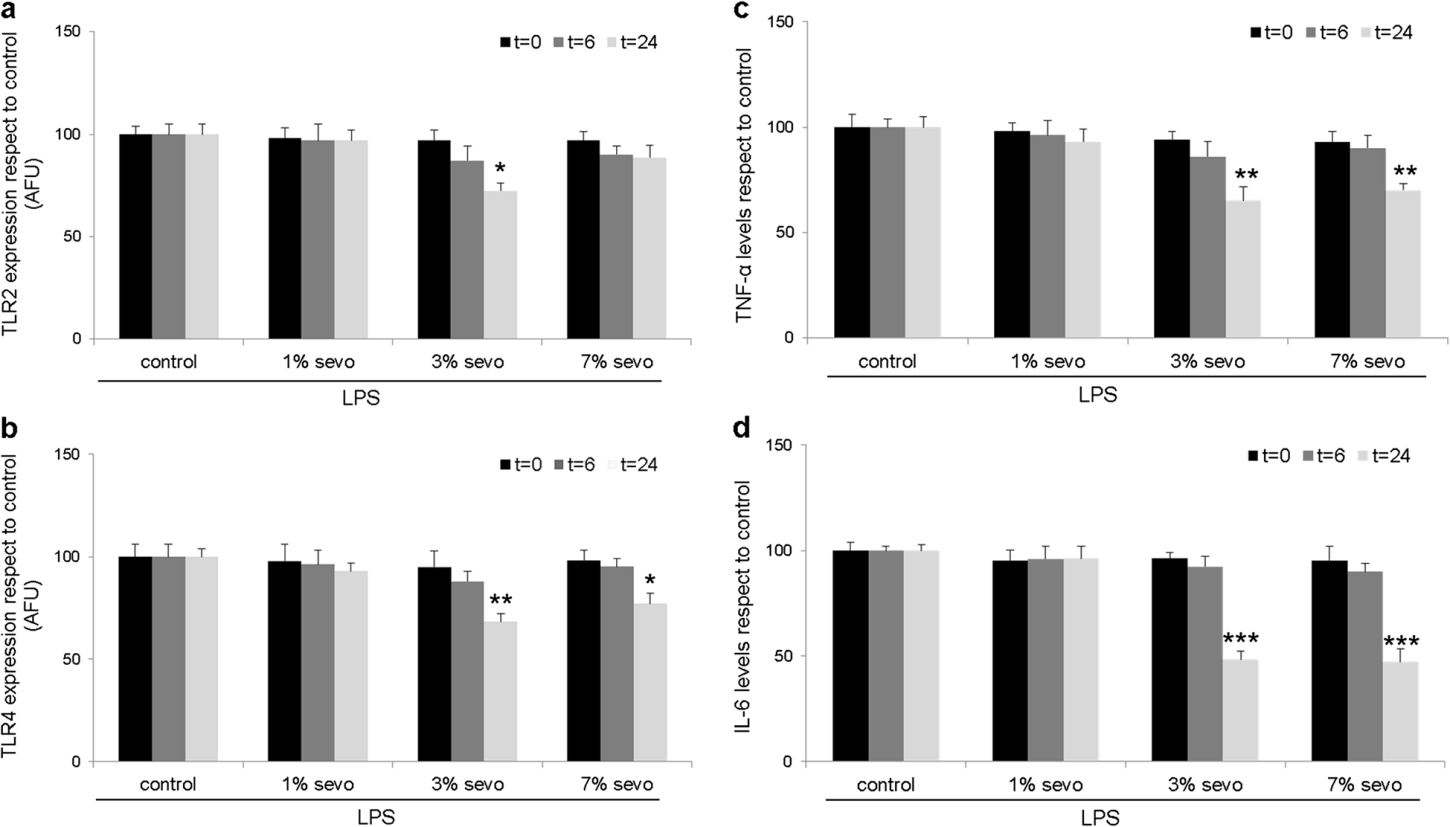
**Effect of sevoflurane postconditioning on HUVEC cultures after LPS stimulation.** (**a**) Effect on TLR2 expression; (**b**) effect on TLR4 expression; (**c**) levels of TNF-α and (**d**) IL-6 after sevoflurane postconditioning on LPS-treated HUVEC cultures. *p <0.05, ** p<0.01, *** p<0.001. AFU: Arbitrary fluorescence units. Analysis was performed immediately after sevoflurane exposure (t=0), at 6 (t=6) and 24 (t=24) hours. LPS: Lipopolysaccharide.

In agreement with previous reports, LPS-treated HUVEC showed a significant increase of TNF-α and IL-6 levels in culture media both at 6 and 24 hours after stimulation (see Additional file 1). The three tested doses of sevoflurane after LPS administration showed different cytoprotective effects on endothelial cultures, as assessed by MTT or Trypan blue assays. Exposure to 1% sevoflurane did not induce a statistically significant cytoprotective effect (Figure 2a,b, all p≥0.05, n=9). Exposure to 3% sevoflurane after LPS lead to a statistically significant cytoprotective effect at 6 and 24 hours compared to control (Figure 2a,b p<0.05 and p<0.01 respectively, n=9), while no significant effect was observed on the 7% sevoflurane group (all p≥0.05, n=9). With respect to TLR expression, exposure to 1% sevoflurane did not change TLR2 or TLR4 levels in our cultures (Figure 3a,b, all p≥0.05, n=9). By contrast, 3% sevoflurane significantly decreased both TLR2 and TLR4 expression 24 hours after exposure compared to control (Figure 3a,b, p<0.05 and p<0.01, respectively, n=9). Exposure to 7% sevoflurane did not affect TLR2 expression (Figure 3a, p≥0.05, n=9), but significantly decreased TLR4 levels at 24 hours (Figure 3b, p<0.05, n=9). Due to the known effect of LPS on cytokine release, we also determined TNF-α and IL-6 levels in this study group. Exposure to 1% sevoflurane did not modify TNF-α or IL-6 levels compared to control (Figure 3c,d, all p≥0.05, n=9). However, 3% and 7% sevoflurane decreased TNF-α (Figure 3c, both p<0.01, n=9) and IL-6 levels (Figure 3d, both p<0.001, n=9) at 24 hours.

## Discussion

The present study shows sevoflurane-induced changes on the inflammatory response in human endothelial cells. Importantly, sevoflurane postconditioning after LPS administration shows a cytoprotective effect, a decrease of TLR2 and TLR4 expression as well as inflammatory mediators.

Sevoflurane has proven to reduce perioperative morbidity and mortality [19,20], showing non-anesthetic properties that involve cytoprotective effects related to its reduction of hypoxia or endotoxaemia-induced cell damage due to mechanisms of pre-, intra- and postconditioning: an exposure to sevoflurane before, during or after an insult. In the context of cardiac surgery [21,22] and cerebral ischemia [23,24], hypoxia-related protective mechanisms of preconditioning with volatile anesthetics include hemodynamic mechanisms, up-regulation of antiapoptotic factors, attenuation of excitotoxicity, or enhancement of endogenous reparative processes in the brain [25,26]. Regarding endotoxaemia, available data from experimental studies suggest that preconditioning with halogenated anesthetics confers protection by inhibition of the inflammatory response [5,27-29].

However, less is known about the postconditioning effect of sevoflurane, a more recent procedure that has also shown to be an effective therapeutic strategy to reduce ischemia-induced damage in the brain and myocardium [30,31] and to attenuate LPS-induced response in experimental models in pulmonary cells [7,32]. These studies have shown that beneficial effects of sevoflurane on LPS-mediated damage involve a reduction of cell death, an attenuation of the inflammatory response and chemotactic mechanisms, as well as an increase in the expression of molecules that protect cells against injury such as the chaperone HSP-32.

TLR2 and TLR4 are potent initiators of the inflammatory and immune response, triggering intracellular signaling cascades that eventually result in the increase of the expression of pro-inflammatory cytokines [33], therefore playing an important role in sepsis pathogenesis [34,35]. Because exposure to sevoflurane has shown to reduce inflammation, we aimed to investigate whether this effect could be mediated by a sevoflurane-induced decrease of TLR expression in the endothelium, as a key tissue involved in sepsis. In non-stimulated endothelial cells, exposure to 3% or 7% sevoflurane decreased TLR4 levels. TLR2 expression was lowered by 3% sevoflurane, but not after exposure to a concentration of 7%, indicating that this is not a dose-dependent effect. In these cultures, the pro-inflammatory mediators TNF-α and IL-6 were also reduced, in agreement with previous studies that reported an anti-inflammatory effect of this anesthetic agent [5,7]. Previous studies have reported an immunomodulatory effect of sevoflurane in mice [36,37], and pulmonary cells [29], without detrimental effects on organ toxicity or cell viability. However, this is, to the best of our knowledge, the first report of a relationship between sevoflurane and TLR expression in human cells.

We subsequently investigated sevoflurane postconditioning effect on LPS-stimulated endothelial cells as an *in vitro* model of endotoxaemia [38], and results indicate that exposure to sevoflurane reduced cell toxicity, TLR2 and TLR4 expression as well as TNF-α and IL-6 levels. This seems to be in accordance with studies by Steurer et.al, reporting sevoflurane-mediated TNF-α decrease in alveolar macrophages after LPS administration [39]. In that study, a significant decrease in TNF-α levels was found as soon as 2 hours after exposure, losing its significance at 24 hours, whereas in our experiments this was the time of maximum attenuation. We think that the different cell type used as well as the exposure length, notably shorter in our study, might account for these differences. With respect to anesthetic concentration, we found the greater biological effects at 3% sevoflurane, similarly to other postconditioning studies [39]. Interestingly, most of our results are also observed at 7% sevoflurane, indicating that its dosage might differentially affect cell types. For example, postconditioning with up to 6% sevoflurane has shown beneficial effects in hippocampal tissue [30].

*In vivo* models of sepsis have demonstrated that pretreatment with sevoflurane decreased TNF-α and IL-6 [28]. Nevertheless, we are not aware of any previous study reporting the postconditioning effect of sevoflurane in endotoxaemia. Our results showing sevoflurane-induced TLR2, TLR4 and cytokine decrease seem to indicate that this protective effect may be also mediated by anti-inflammatory mechanisms. Until what extent the observed decrease of cytokine levels is due to TLR inhibition is not explored in this preliminary study. Given the important role of TLR on cytokine production via NF-ĸb activation, it is possible that lower levels of TNF-α and IL-6 might be, at least, partially explained by a decrease in TLR expression. However, a direct causal effect between TLR and cytokines cannot be concluded from our experiments, but only to hypothesize about a possible biological association that should be further explored.

Importantly, in agreement with previous studies [40,41], TLR2 expression was low in our endothelial cells, becoming higher after LPS stimulation. In this regard, we cannot exclude the possibility that this activation might be, at least in part, due to the presence of small amounts of lipoproteins in LPS preparation. Also of note, an increase of TLR2 expression in endothelial cells under inflammatory conditions has also been demonstrated, existing an important synergic effect between LPS, TNF-α and TLR2 expression [42-44]. Due to this complex scenario, we think that TLR2 levels should be cautiously interpreted.

The ability of sevoflurane to modulate different immunity pathways and its potential role as an immunomodulatory drug in critically ill patients, expand its therapeutic value beyond anesthetic properties. The present study suggests that this volatile anesthetic might help to preserve endothelium integrity in severe endotoxaemia. Though caution should be exercised, it is tempting to postulate that sevoflurane might offer a therapeutic value in septic patients. In order to attenuate inflammatory response and reduce organ failure, targeting TLR signaling has been proposed as a therapeutic strategy in sepsis [35].

A minor limitation of this study is that, although widely used as a model, stimulation of endothelial cells with LPS does not completely replicate sepsis. In this regard, future *in vivo* studies might help to clarify the effect of sevoflurane postconditioning in sepsis. In addition, because TLRs are major components of the innate immune system, the potential effect of sevoflurane on their expression should be also analyzed in other cell types such as monocytes/macrophages or dendritic cells.

## Conclusions

Our results show that postconditioning with sevoflurane using an experimental *in vitro* model of sepsis exerts protective effects on cell viability, reducing TLR2 and TLR4 expression as well as inflammatory markers in human endothelial cells. More extensive *in vitro* and *in vivo* studies exploring the ability of sevoflurane postconditioning to modify immunity and inflammatory pathways are required to elucidate its potential value in the clinical setting.

## Competing interests

The authors declare that they have no competing interests.

## Authors’ contributions

RRG, AB and JAL have conceived and designed the research; analyzed and interpreted the data; performed statistical analysis, and drafted the manuscript. SVDR, AR, JR, MT and DB analyzed and interpreted the data and made critical revision of the manuscript. All authors read and approved the final manuscript.

## Supplementary Material

Additional file 1: Figure S1 Scheme showing experimental plan for HUVEC cultures exposed to sevoflurane **(a)** and sevoflurane postconditioning after LPS exposure **(b)**. t=0: samples taken immediately after exposure, t=6, t=24: samples taken 6 and 24 hours after exposure, respectively. **Figure S2.** HUVEC stimulated with a concentration of 1 μg/mL LPS showed a significant increase of TNF-alpha; and IL-6 levels in culture media. Control: unstimulated cells; * p <0.05, ** p <0.001. **Figure S3.** Exposure of HUVEC cultures to sevoflurane did not increase cell death measured by MTT (**a**) or Trypan blue (**b**) assays at different timepoints (all p≥0.05, n=9). Click here for file
