# Supplementary material for: Effects of sevoflurane postconditioning on cell death, inflammation and TLR expression in human endothelial cells exposed to LPS
Source: J Transl Med. 2013 Apr 3;11:87. doi: 10.1186/1479-5876-11-87 (PMC3636049; doi:10.1186/1479-5876-11-87)
Supplement: Additional file 1: Figure S1 — Scheme showing experimental plan for HUVEC cultures exposed to sevoflurane (a) and sevoflurane postconditioning after LPS exposure (b). t=0: samples taken immediately after exposure, t=6, t=24: samples taken 6 and 24 hours after exposure, respectively. Figure S2. HUVEC stimulated with a concentration of 1 μg/mL LPS showed a significant increase of TNF-alpha; and IL-6 levels in culture media. Control: unstimulated cells; * p <0.05, ** p <0.001. Figure S3. Exposure of HUVEC cultures to sevoflurane did not increase cell death measured by MTT (a) or Trypan blue (b) assays at different timepoints (all p≥0.05, n=9). [file 1479-5876-11-87-S1.pdf]

**Figure 1**

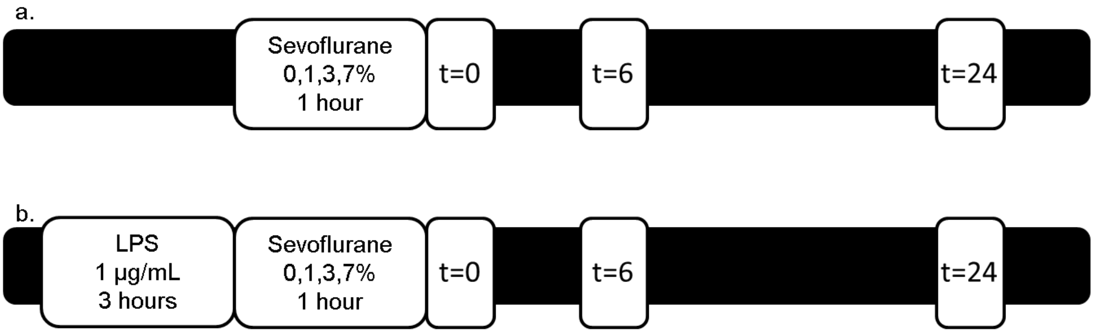


Scheme showing experimental plan for HUVEC cultures exposed to sevoflurane **(a)** and sevoflurane postconditioning after LPS exposure **(b)**. t=0: samples taken immediately after exposure, t=6, t=24: samples taken 6 and 24 hours after exposure, respectively.

**Figure 2**

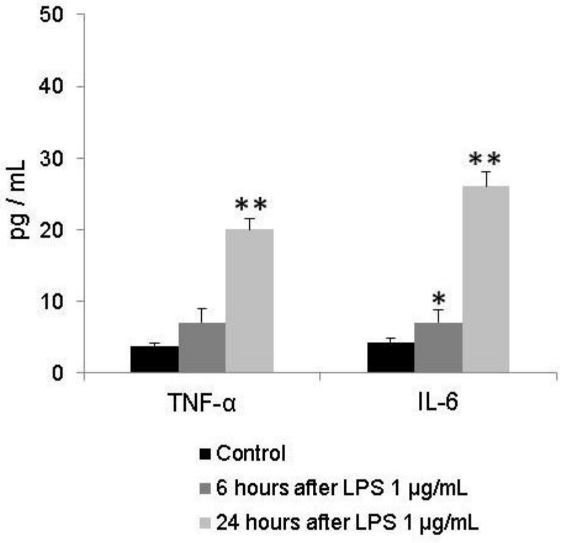


HUVEC stimulated with a concentration of 1 µg/mL LPS showed a significant increase of TNF-α and IL-6 levels in culture media. Control: unstimulated cells; * p <0.05, ** p <0.001.

**Figure 3**

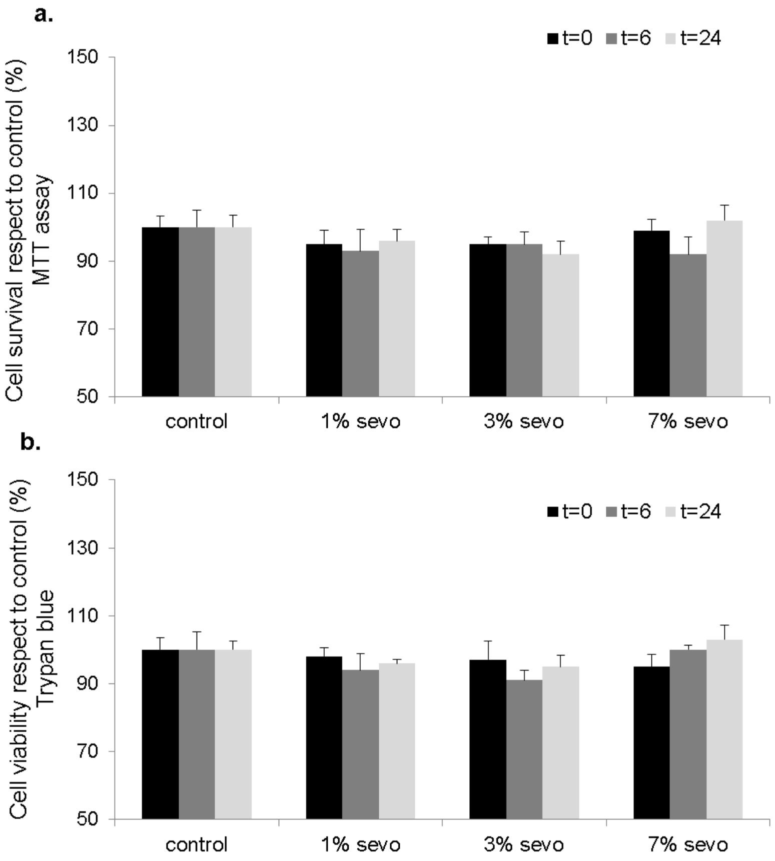


Exposure of HUVEC cultures to sevoflurane did not increase cell death measured by MTT **(a)** or Trypan blue **(b)** assays at different timepoints (all $p \geq 0.05$, $n=9$).
